# Genome-Wide Identification of the A20/AN1 Zinc Finger Protein Family Genes in *Ipomoea batatas* and Its Two Relatives and Function Analysis of *IbSAP16* in Salinity Tolerance

**DOI:** 10.3390/ijms231911551

**Published:** 2022-09-30

**Authors:** Hao Xie, Qiangqiang Yang, Xiaoxiao Wang, Michael R. Schläppi, Hui Yan, Meng Kou, Wei Tang, Xin Wang, Yungang Zhang, Qiang Li, Shaojun Dai, Yaju Liu

**Affiliations:** 1 Key Laboratory of Saline-Alkali Vegetation Ecology Restoration, Ministry of Education, College of Life Sciences, Northeast Forestry University, Harbin 150040, China; 2 Xuzhou Institute of Agricultural Sciences in Jiangsu Xuhuai District/Jiangsu Xuzhou Sweetpotato Research Center/Key Laboratory of Biology and Genetic Improvement of Sweetpotato, Ministry of Agriculture and Rural Affairs/Sweetpotato Research Institute, Chinese Academy of Agricultural Sciences, Xuzhou 221131, China; 3 Department of Biological Sciences, Marquette University, Milwaukee, WI 53233, USA; 4 Development Center of Plant Germplasm Resources, College of Life Sciences, Shanghai Normal University, Shanghai 200234, China

**Keywords:** sweetpotato, stress-associated protein (SAP), A20/AN1 zinc finger, IbSAP, abiotic stress

## Abstract

Stress-associated protein (SAP) genes—encoding A20/AN1 zinc-finger domain-containing proteins—play pivotal roles in regulating stress responses, growth, and development in plants. They are considered suitable candidates to improve abiotic stress tolerance in plants. However, the *SAP* gene family in sweetpotato (*Ipomoea batatas*) and its relatives is yet to be investigated. In this study, 20 *SAPs* in sweetpotato, and 23 and 26 *SAPs* in its wild diploid relatives *Ipomoea triloba* and *Ipomoea trifida* were identified. The chromosome locations, gene structures, protein physiological properties, conserved domains, and phylogenetic relationships of these *SAPs* were analyzed systematically. Binding motif analysis of *IbSAPs* indicated that hormone and stress responsive *cis*-acting elements were distributed in their promoters. RT-qPCR or RNA-seq data revealed that the expression patterns of *IbSAP*, *ItbSAP*, and *ItfSAP* genes varied in different organs and responded to salinity, drought, or ABA (abscisic acid) treatments differently. Moreover, we found that *IbSAP16* driven by the *35 S* promoter conferred salinity tolerance in transgenic *Arabidopsis*. These results provided a genome-wide characterization of *SAP* genes in sweetpotato and its two relatives and suggested that *IbSAP16* is involved in salinity stress responses. Our research laid the groundwork for studying SAP-mediated stress response mechanisms in sweetpotato.

## 1. Introduction

Stress-associated protein (SAP) genes, encoding A20/AN1 zinc-finger domain-containing proteins, are considered good candidates to improve abiotic stress tolerance in crops [1,2]. The first *SAP* gene identified in plants was *OsiSAP1* from elite *indica* rice (*Oryza sativa* L. var. Pusa Basmati-1) [3]. Members of this family contain one or two types of special zinc-finger domains, A20 and/or AN1. The A20 domain was first found in the A20 protein from human umbilical vein endothelial cells, and its conserved structure is CX_2-4_CX_11_CX_2_C (C stands for cysteine residue and X stands for other amino acid residues) [4]. The AN1 domain was first identified in the protein encoded by *animal hemisphere 1* (*AN1*) from *Xenopus laevis* [5], and its conserved structure in plants is CX_2/4_CX_9-12_CX_1-2_CX_4_CX_2_HX_5_HXC (H stands for histidine residue) [6].

It has been shown that many *SAP* genes are involved in abiotic stress responses in plants. Overexpression or ectopic expression of most *SAPs* enhanced stress tolerance in transgenic plants. For example, ectopic expression or overexpression of *OsiSAP1/OsSAP11* conferred tolerance to multiple stresses in transgenic tobacco [3], *Arabidopsis* [7], or rice [8]. *AtSAP5*-overexpressing plants showed stronger drought tolerance than wildtype (WT) plants [9,10]. Overexpression of *AtSAP13* enhanced salinity, drought, and heavy metal tolerance [11]. *OsSAP7*-overexpressing rice plants were insensitive to drought, low temperature, and salinity stresses [12]. However, not all *SAPs* are positive regulators of stress responses in plants. Mutants of *AtSAP9* had higher germination rates than WT *Arabidopsis* under abscisic acid (ABA) treatment, while the germination rates of *AtSAP9*-overexressing plants was lower than that of WT plants [13].

SAPs are involved in biotic stress responses as well. Ectopic expression of *OsSAP1* upregulated defense-related genes and enhanced bacterial pathogen resistance of transgenic tobacco [14]. Overexpression of *AtSAP9* reduced *Pseudomonas syringae* pv. *phaseolicola* resistance of *A. thaliana* [13]. Overexpression or ectopic expression of *AtSAP5* or its ortholog in *P. aphrodite*, *Pha13,* increased virus resistance of transgenic plants, and *AtSAP5-*RNAi plants were more susceptible to virus infection than wild-type plants [15]. Silencing of *SlSAP3* in tomato (*Solanum lycopersicum*) decreased the resistance to *Pseudomonas syringae* pv. tomato (*Pst*) DC3000 of tomato, while overexpression of *SlSAP3* enhanced the resistance [16].

To date, *SAP* family members have been identified and characterized in rice (*Oryza sativa*), *Arabidopsis thaliana* [2], maize (*Zea mays*), *Populus trichocarpa* [6], tomato (*Solanum lycopersicum*) [17], cotton (*Gossypium hirsutum*) [18], *Medicago truncatula* [19], apple (*Malus* × *domestica*) [20], *Brassica napus* [21], soybean (*Glycine max*) [22], and cucumber (*Cucumis sativus*) [23]. However, little is known about the *SAP* gene family in sweetpotato (*Ipomoea batatas* (L.) Lam., 2n = 6x = 90).

Sweetpotato is an important food crop in the world, mainly used for human food, animal feed, and manufacturing starch and its products [24]. The production of sweetpotato suffers yield loss due to drought and salinity stresses. Therefore, it is important to improve salinity and drought tolerance of sweetpotato. Some stress-tolerant genes have been identified, such as *IbABF4* [25], *IbINH* [26], *IbSUT4* [27], and *IbBBX-IbTOE3-IbPRX* module [28]. The sweetpotato genome has been sequenced and assembled into 15 pseudo-chromosomes [29]. Although this genome was not precisely reconstructed, it has laid the groundwork for gene identification, cloning, and functional research in sweetpotato. To identify more candidate genes for molecular breeding to improve abiotic stress tolerance of sweetpotato, we screened *SAP* genes in sweetpotato and its two wild relatives and analyzed their gene structures, conserved domains, chromosome localization, phylogenetic relationships, *cis-*elements in promoter regions, and expression patterns. Moreover, we primarily investigated the function of *IbSAP16*, which encodes an SAP protein with two AN1 and two C_2_H_2_ domains. To date, there is only one report about the function of an *IbSAP16* ortholog—*AtSAP13—*which conferred tolerance to multiple abiotic stresses [11]. Our results indicated that *IbSAPs* are involved in stress responses in sweetpotato, and *IbSAP16* enhanced salinity tolerance in transgenic *Arabidopsis*. Our research laid a foundation for illuminating mechanisms of *SAP*-mediated stress responses in sweetpotato.

## 2. Results

### 2.1. Identification of SAPs in Sweetpotato and Its Two Diploid Wild Relatives

To identify *SAP* genes in sweetpotato, a scan was carried out using the hidden Markov models of A20/AN1 zinc fingers against the sweetpotato genome [29] and transcriptome databases (unpublished data). A total of 38 candidate sequences were obtained. Based on CD-search results, 20 of them were validated as *IbSAP* members and named as *IbSAP1* through *IbSAP20*. Most *IbSAP* genes were identified in both the genome and transcriptome, but *IbSAP13* and *IbSAP17* were only identified in the transcriptome. *IbSAP4*, *IbSAP11*, and *IbSAP18* had two copies, while the others had only one copy. Coding sequence (CDS) lengths of *IbSAPs* ranged from 429 bp (*IbSAP8*) to 885 bp (*IbSAP17*), while the molecular weight (Mw) of IbSAP proteins ranged from 15.40 kDa (IbSAP20) to 32.56 kDa (IbSAP17). The predicted grand average of hydropathicity (GRAVY) index of IbSAPs ranged from −0.935 (IbSAP9) to −0.346 (IbSAP2), and the isoelectric point (pI) distribution of IbSAPs was from 7.49 (IbSAP4 and IbSAP5) to 9.48 (IbSAP14), which indicated that IbSAPs are hydrophilic alkaline proteins (GRAVY < 0 and pI > 7). LOCTREE 3 website analysis suggested that most IbSAPs were located in the cytoplasm (IbSAP1–14, IbSAP18–20); however, IbSAP15 and IbSAP16 might be located in the endoplasmic reticulum and IbSAP17 in the nucleus (Table 1).

We also screened the genomes of *Ipomoea triloba* and *Ipomoea trifida,* two relative species of sweetpotato [30] and identified 23 and 26 *SAP* genes, respectively. They were named *ItbSAP/ItfSAP1–22(L) (Like)* according to their phylogenetic relationship with *IbSAP*s (Table S1). Among these *SAP* genes, *ItbSAP7*, *ItbSAP18*, *ItbSAP22*, *ItfSAP7L*, *ItfSAP18*, and *ItfSAP22* were multicopy genes. Each IbSAP and their orthologs in *I. triloba* and *I. trifida* share similar amino acid sequences with a coverage ranging from 98.01% to 100% and a gap percentage ranging from 0.00% to 3.98%. Orthologs from the three *Ipomoea* species share a high degree of identity (90.73% to 100%) and positive amino acids (92.72% to 100%) (Table S2). These results indicated that SAPs are highly conserved in *I. batatas* and its wild relatives, *I. triloba* and *I. trifida*. We did not find any orthologs of two *ItbSAP*s (*ItbSAP21/22*) and two *ItfSAP*s (*ItfSAP21/22*) in the sweetpotato genome and/or transcriptome. *IbSAP18*, *ItbSAP18*, and *ItfSAP18* had two gene copies each (Table 1 and Table S1), which indicated that *SAP18* duplicated in the two relatives or their ancestor(s).

**Figure 1:**
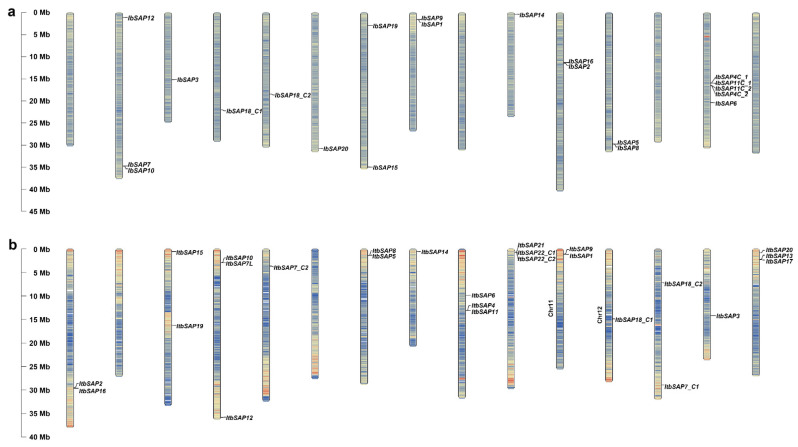

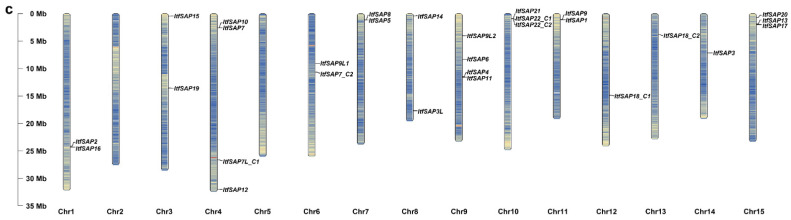
Chromosome localization of *SAP* genes in *I. batatas* (**a**), *I. triloba* (**b**), and *I. trifida* (**c**). The bars on the left represent the length of chromosomes. The chromosome numbers and gene names are displayed on the left side and right side of chromosomes, respectively.

Subsequently, the distribution of *IbSAP* genes on the 15 pseudo-chromosomes in sweetpotato genome was analyzed (Figure 1a). *ItbSAPs* and *ItfSAPs* were also mapped onto the chromosomes of *I. triloba* and *I. trifida*, respectively (Figure 1b,c). We noticed that the distribution of *IbSAPs* and *Itb/ItfSAPs* are similar (Figure 1). For example, *IbSAP7*, *IbSAP10*, and *IbSAP12* are located on chromosome 2 (Chr2) of sweetpotato; while *ItbSAP7L*, *ItbSAP10*, and *ItbSAP12,* as well as *ItfSAP7*, *ItfSAP10*, and *ItfSAP12* are located on Chr4 of *I. triloba* and *I. trifida*, respectively. Similar distribution of *SAPs* in sweetpotato and its two diploid relatives were also observed on the *I. batatas* Chr3 (IbChr3) and Ch14 of *I. triloba* and *I. trifida* (ItbChr14 and ItfChr14) (*Ib/Itb/ItfSAP3*), IbChr4 and Itb/ItfChr12 (*Ib/Itb/ItfSAP18_C1*), IbChr5 and Itb/ItfChr13 (*Ib/Itb/ItfSAP18_C2*), IbChr7 and Itb/ItfChr3 (*Ib/Itb/ItfSAP15* and *19*), IbChr8 and Itb/ItfChr11 (*Ib/Itb/ItfSAP1* and *9*), IbChr10 and Itb/ItfChr8 (*Ib/Itb/ItfSAP14*), IbChr11 and Itb/ItfChr1 (*Ib/Itb/ItfSAP2* and *16*), IbChr12 and Itb/ItfChr7 (*Ib/Itb/ItfSAP5* and *8*), and IbChr14 and Itb/ItfChr9 (*Ib/Itb/ItfSAP4*, *6*, and *11*). *Ib/Itb/ItfSAP20* are located on IbChr6, ItbChr15, and ItfChr15, respectively. Moreover, *Itb/ItfSAP13* and *Itb/ItfSAP17* are located on ItbChr15/ItfChr15 along with *Ib/Itb/ItfSAP20*, while *IbSAP13* and *IbSAP17* were not detected in the sweetpotato genome, but were identified in the Xu28 transcriptome. We inferred that *IbSAP13* and *IbSAP17* are located on IbChr6 along with *IbSAP20*, but for some reason could not be detected. In addition, *ItbSAP7* is located on Chr5 (*ItbSAP7_C2*) and Chr13 (*ItbSAP7_C1*), not on the corresponding chromosome that harbored *IbSAP7* (IbChr2) and *ItfSAP7* (ItfChr4) (Figure 1), suggesting that *IbSAP7* may have evolved from *ItfSAP7*, but not *ItbSAP7*.

### 2.2. Phylogenetic Analysis of IbSAPs and SAPs from Some Other Plants

**Figure 2:**
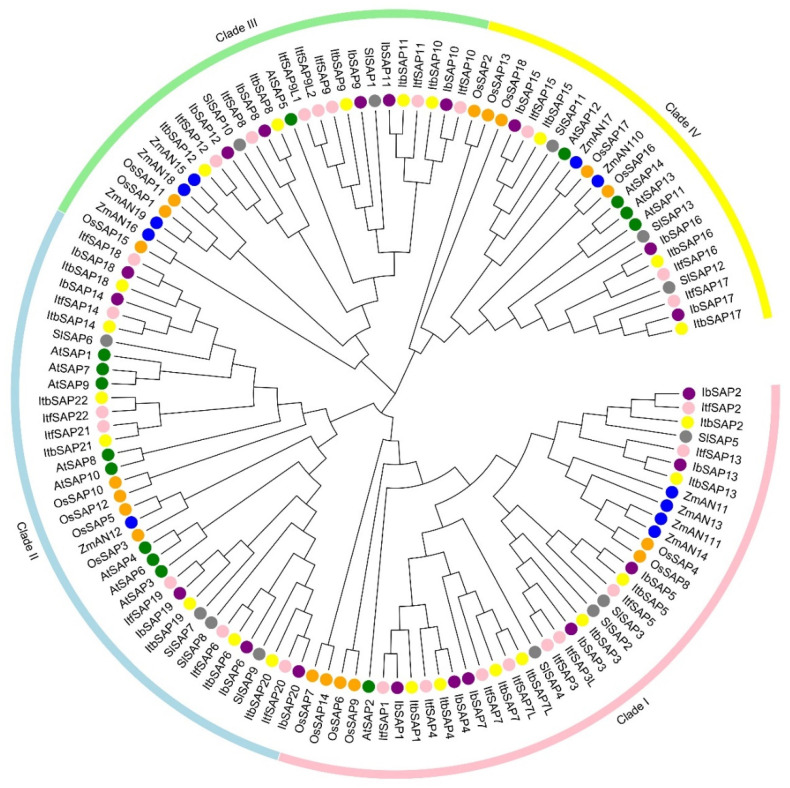
Phylogenetic relationship of stress-associated proteins (SAPs) from *I. batatas*, *I. triloba*, *I. trifida, Arabidopsis thaliana*, *Oryza sativa*, *Solanum lycopersicum*, and *Zea mays*. A total of 125 SAPs were divided into four clades (clade I to IV). The cycles filled with purple, yellow, pink, green, orange, grey, and blue represent SAPs in *I. batatas* (IbSAPs), *I. triloba* (ItbSAPs), *I. trifida* (ItfSAPs), *A. thaliana* (AtSAPs), *O. sativa* (OsSAPs), *S. lycopersicum* (SlSAPs), and *Z. mays* (ZmAN1s), respectively. The accession numbers of the sequences used in this phylogenetic analysis are listed in Table 1 and Table S1.

To investigate the phylogenetic relationship between IbSAPs and their orthologs in the two relative species of sweetpotato as well as other plants, a total of 125 SAP protein sequences were used to build an unrooted neighbor-joining phylogenetic tree (Figure 2). Similar to the phylogenetic relationship of 14 AtSAPs and 18 OsSAPs reported before [2], all SAPs examined fall into four clades. In each clade, numbers of SAPs in each species were as follows (total: *I. batatas*, *I. triloba*, *I. trifida*, *A. thaliana*, *O. sativa*, *S. lycopersicum*, *Z. mays*): clade I (7, 8, 9, 1, 6, 4, 4); clade II (5, 7, 7, 8, 4, 1, 4); clade III (5, 5, 7, 1, 3, 4, 2); clade IV (3, 4, 3, 4, 5, 2, 3). Each specific clade included SAPs of both monocotyledons (*O. sativa* and *Z. mays*) and dicotyledons (*I. batatas*, *I. triloba*, *I. trifida*, *A. thaliana*, and *S. lycopersicum*) plants. However, some subclades were monocot specific (such as ZmAN11 to OsSAP8 in clade I or ZmAN15 to OsSAP15 in clade III) or dicot specific (such as SlSAP4 to ItfSAP1 in clade I or ItfSAP10 to ItbSAP12 in clade III). These results indicated that SAP proteins appeared before the divergence of monocot and dicot plants and have been evolving after species divergence. The number and distribution of IbSAPs was most similar to the two wild relative species. However, there were more SAP members in *I. triloba* and *I. trifida* than in *I. batatas*. As mentioned above, no SAPs in *I. batatas* were orthologous to ItfSAP21/22 and ItbSAP21/22. Moreover, two or more sequences in *I. trifida* or *I. triloba* were orthologous to certain IbSAP members. Two sequences (ItfSAP3 and ItfSAP3L) in *I. trifida* were orthologous to IbSAP3. Two sequences (ItfSAP7 and ItfSAP7L) in *I. trifida* and two sequences (ItbSAP7 and ItbSAP7L) in *I. triloba* were orthologous to IbSAP7. Three sequences (ItfSAP9, ItfSAP9L1, and ItfSAP9L2) in *I. trifida* were orthologous to IbSAP9 (Figure 2). That fewer SAPs were identified in sweetpotato than in its two diploid relatives might be due to some alteration in chromosome features that occurred during or after the evolution of sweetpotato, or due to the incomplete sweetpotato genome sequence.

### 2.3. Gene and Protein Structures of IbSAPs, ItbSAPs, and ItfSAPs

**Figure 3:**
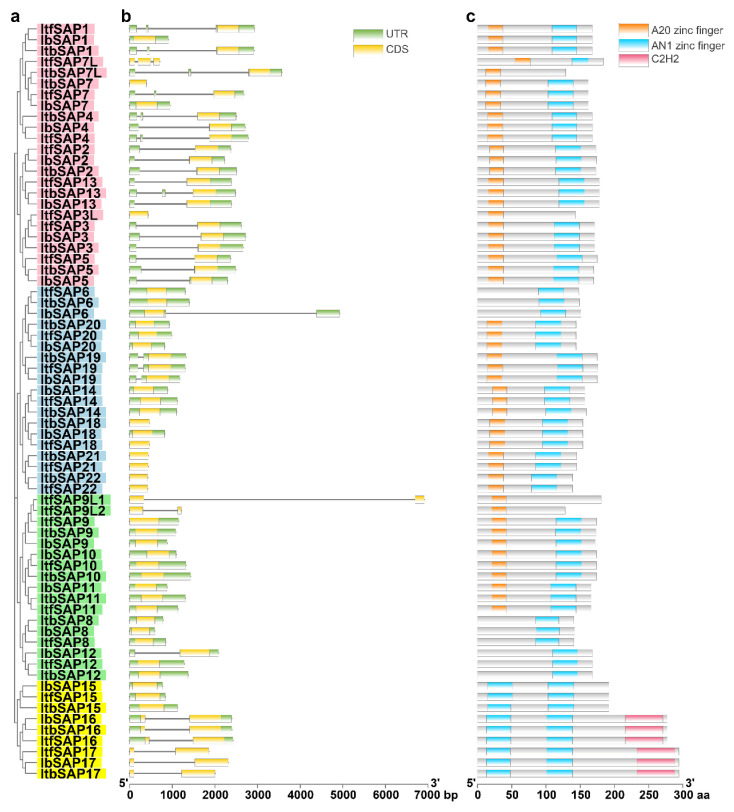
Gene structure and conserved domain analysis of IbSAPs, ItbSAPs, and ItfSAPs. The phylogenetic tree (**a**) was built by MEGA-X via the neighbor-joining method for 1000 bootstrap replicates using the full-length amino acid sequence alignment by the MUSCLE algorithm. Gene structures (**b**) obtained from the annotation of *I. batatas*, *I. triloba*, and *I. trifida* genome and conserved domains (**c**) analyzed by CD-search were visualized using tbTools.

The gene structures of *Ib/Itb/ItfSAPs* (Figure 3b) and conserved domains of their encoding proteins (Figure 3c and Figure S1) were analyzed sorted based on the phylogenetic tree of IbSAPs, ItbSAPs, and ItfSAPs (Figure 3a). The gene structures of *IbSAPs* were similar to those of *ItbSAPs* and *ItfSAPs*, and were conserved in each clade. In clade I, most *SAPs* have one or two introns in their 5′-untranslated regions (5′-UTRs), except that *IbSAP1*, *ItfSAP3L*, *IbSAP7*, and *ItbSAP7* have no intron, and that *ItfSAP7L* has two introns in the coding sequence. In clades II and III, most *SAPs* have no intron, except that *IbSAP6* has one intron in the 3′-UTR, *Ib/Itb/ItfSAP19* and *IbSAP12* have one intron in the 5′-UTR, and that *ItfSAP9L1/2* have one intron in the coding sequence. In clade IV, *Ib/Itb/ItfSAP15* have no intron, while *Ib/Itb/ItfSAP16/17* have one intron in coding sequences.

Protein structure analysis showed that SAPs from *I. batatas*, *I. triloba*, and *I. trifida* in each clade were conserved. Most Ib/Itb/ItfSAPs in clades I, II, and III had one A20 zinc finger and one AN1 zinc finger (Figure 3c and Figure S1), which is considered a “typical SAP” [1]. Ib/Itb/ItfSAP8, and Ib/Itb/ItfSAP12, only had an AN1 domain (Figure 3c). We found an atypical A20 domain in Ib/Itb/ItfSAP6 (CX_3_CX_8_CX_2_C) (Figure S1), while a typical A20 domain should be CX_2-4_CX_11_CX_2_C [4]. This kind of variation only occurred in Ib/Itb/ItfSAP6 out of all SAPs mentioned in this study. Moreover, we found just one conserved cysteine residue instead of an A20 domain in Ib/Itb/ItfSAP8 and Ib/Itb/ItfSAP12 (Figure S1). In addition, some SAP proteins in *I. triloba* and *I. trifida* only had an A20 domain and no AN1 domain, such as ItbSAP7L, ItfSAP3L, and ItfSAP9L1/2 (Figure 3c). These SAPLs might have evolved due to mutations after gene duplication. In addition, clade IV Ib/Itb/ItfSAPs had only two AN1 domains as well as 0/2 C_2_H_2_ domains (Figure 3c). The similar gene structures and conserved protein domains in each clade indicate that Ib/Itb/ItfSAPs in each clade might have expanded due to gene duplication.

A20 domains in SAPs of the three *Ipomoea* species analyzed were conserved (except for the incomplete A20 domain in Ib/Itb/ItfSAP6), while AN1 domains differed among them. The AN1 domain sequence in typical Ib/Itb/ItfSAPs with both A20 and AN1 domains was CX_2_CX_10_CX_1_CX_4_CX_2_HX_5_HXC, which is considered a typical AN1 domain pattern (CX_2/4_CX_9-12_CX_1-2_CX_4_CX_2_HX_5_HXC). The AN1 domain sequence in Ib/Itb/ItfSAP8 and Ib/Itb/ItfSAP12 (containing only one AN1 domain) was CX_4_CX_10_CX_1_CX_4_CX_2_HX_5_HXC, and in Ib/Itb/ItfSAP15–17 (containing only two AN1 domains and 0/2 C_2_H_2_ domains) was CX_4_CX_12_CX_2_CX_4_CX_2_HX_5_HXC (Figure S1), which is considered an expanded pattern of AN1 [6]. A similar variant AN1 domain was also found in the SlSAP10 protein of tomato, SX_4_CX_10_CX_1_CX_4_CX_2_HX_5_HXC (S stands for serine residue), in which the first cysteine residue is substituted by a serine residue (Figure S1). Considering sweetpotato and tomato belong to the Solanales, this result suggests that a specific evolution event occurred in SAP families of Solanales.

### 2.4. Cis-Acting Elements in the Promoter of IbSAPs

To predict regulatory factor binding sites in promoters of *IbSAP* genes, 2-kb upstream sequences of *IbSAPs* were analyzed. The *cis*-acting elements identified in *IbSAP* promoters can be divided into three groups, i.e., hormone response elements, stress response elements, and light signal response elements (Table 2, Figure S2). Hormone response elements in the promoters included ABRE, as-1, ERE, GARE-motif, P-box, TCA-element, and TGA-box, which mainly respond to ABA, auxin, salicylic acid, methyl jasmonate, ethene, or gibberellin (Table 2). Stress response elements included DRE, LTR, MBS, MYB, STRE, TC-rich repeats, W box, WRE3, and WUN-motif, which are mainly involved in defense and stress responses. Light signal response elements included Box 4, TCT-motif, Sp1, chs-CMA1a/2a, GATA-motif, G-Box, and GT1-motif, which are involved in light responses. Distribution of *cis*-acting elements in *IbSAP* promoters are shown in Figure S2. This result indicated that *IbSAP* genes are primarily regulated by hormones, stresses, and light signals.

### 2.5. Expression Patterns in Different Organs of IbSAPs, ItbSAPs, and ItfSAPs

**Figure 4:**
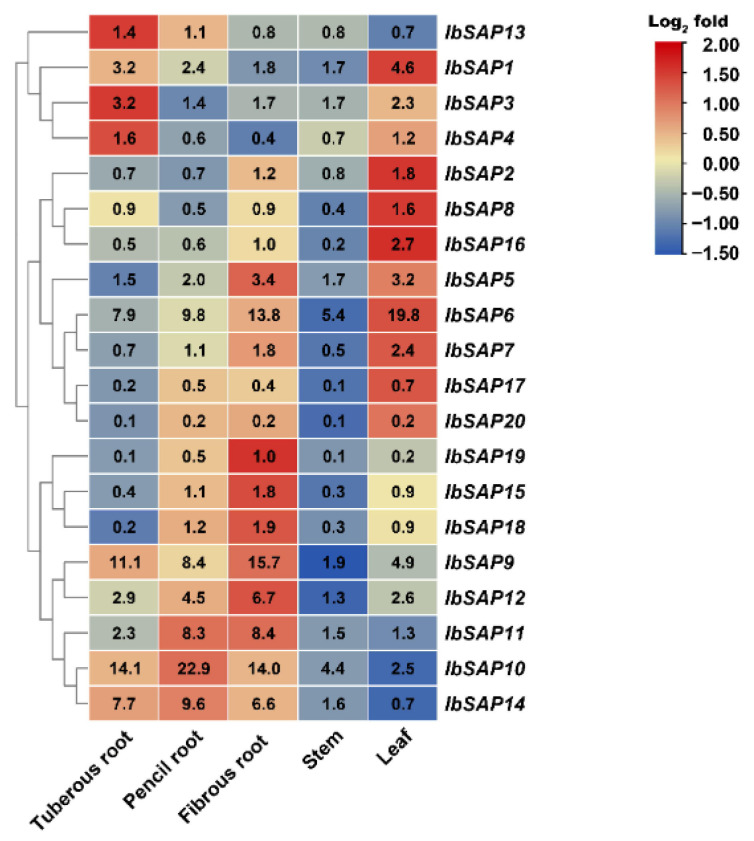
Expression profiles of *IbSAP* genes in different sweetpotato organs. The expression levels of *IbSAPs* were determined by RT-qPCR and quantified using the 2^−ΔΔCt^ method. *I. batatas ADP-ribosylation factor* (*IbARF*, JX177359) was used as an internal reference. The mRNA level of *IbSAP16* in fibrous roots was set as 1 and used to normalize the expression levels of other *IbSAP* genes.

To investigate expression patterns of *IbSAPs* in different organs, *IbSAP* mRNA levels in leaf, stem, tuberous root, pencil root, and fibrous root of 90-day-old sweetpotato plants were analyzed (Figure 4). Based on their mRNA profiles, the 20 *IbSAP*s were divided into three groups. The first group—including *IbSAP1*, *3*, *4*, and *13—*had the highest or second-highest expression levels in tuberous roots, indicating that these *IbSAPs* might function during the development of tuberous root. The second group—including *IbSAP2*, *5–8*, *16*, *17*, and *20—*had the highest or second-highest expression levels in leaves, indicating that these *IbSAPs* might function in photosynthesis, immunology, or leaf development. The third group, including the other *IbSAPs*, had the highest expression levels in pencil roots or fibrous roots, indicating that these *IbSAPs* might function in absorption and utilization of nutrients and water. Besides, most *IbSAP*s had the lowest or second-lowest mRNA levels in stems.

**Figure 5:**
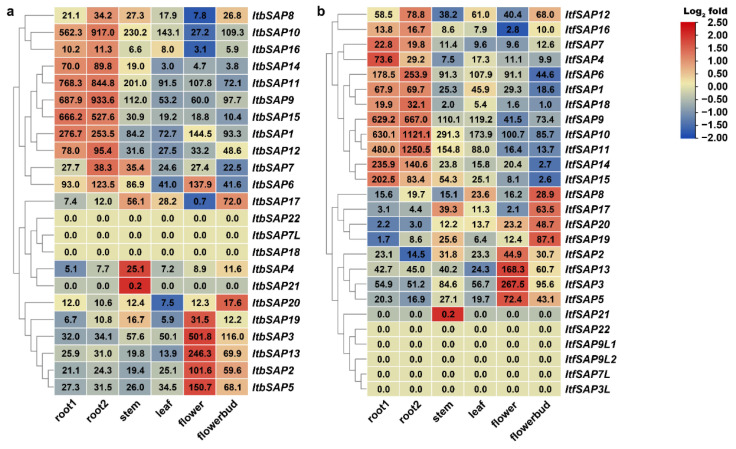
Gene expression patterns of *ItbSAP*s (**a**) and *ItfSAP*s (**b**) in different organs (root 1, root 2, stem, leaf, flower, and flower bud) as determined by RNA-seq. FPKM is shown in the boxes.

Organ-specific expression patterns of *SAPs* in the two diploid relatives of sweetpotato were also analyzed using the RNA-seq results of root 1, root 2, stem, leaf, flower, and flower bud of *I. triloba* and *I. trifida* [30]. The *Itb/ItfSAPs* were divided into two groups according to their organ-specific expression patterns (Figure 5). The first group included *Itb/ItfSAPs* highly expressed in roots. In *I. triloba*, the first group included *ItbSAP1*, *6–12*, and *14–16*; while in *I. trifida*, the first group included *ItfSAP1, 4*, *6*, *7*, *9–12*, *14–16*, and *18*. The second group included *Itb/ItfSAPs* highly expressed in aerial parts (i.e., stem, leaf, flower, or flower bud) or were not expressed in the organs examined. *ItbSAP4*, *ItbSAP21*, and *ItfSAP21* had the highest expression level in stems while *ItbSAP21* and *ItfSAP21* were only detected in stems. Two *ItbSAPs* (*ItbSAP17* and *20*) and four *ItfSAPs* (*ItfSAP8*, *17*, *19*, and *20*) had highest expression levels in flower buds, while five *ItbSAP4s* (i.e., *ItbSAP2*, *3*, *5*, *13*, and *19*) and four *ItfSAPs* (*ItfSAP2*, *3*, *5*, and *13*) had the highest expression levels in flowers. Here, *ItbSAP8* (expressed more in root than in leaf) and *ItfSAP8* (expressed more in leaf than in root) had different expression patterns, and the expression pattern of *ItfSAP8* was more similar to that of *IbSAP8*, showing highest expression levels in leaves (Figure 4). In addition, three *ItbSAPs* (*ItbSAP7L*, *18*, and *22*) and five *ItfSAPs* (*ItfSAP3*, *7*, *9L1*, and *9L2*) were not detected in any organs examined.

### 2.6. Expression Patterns of IbSAPs, ItbSAPs, and ItfSAPs in Response to Salinity, Drought, or ABA Treatments

**Figure 6:**
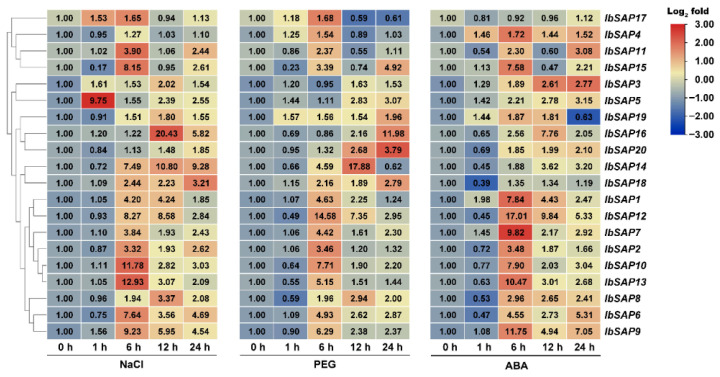
Expression patterns of *IbSAP* genes in response to salinity, drought, and ABA treatment. Sweetpotato shoots were treated with 100 mmol·L^−1^ NaCl, 15% polyethylene glycol (PEG), or 10 μmol·L^−1^ ABA, and the mRNA levels were quantified by RT-qPCR and normalized using the 2^−ΔΔCt^ method. *IbARF* (JX177359) was used as an internal reference.

Since the promoter regions of *IbSAP*s had stress response and hormone response *cis*-acting elements, we determined whether *IbSAP*s responded to salinity, drought, or ABA treatments (Figure 6). Although *IbSAP4* was insensitive to salinity and *IbSAP17* insensitive to salinity and ABA treatments, most *IbSAP*s were induced within 6 h after salinity, drought, or ABA treatments, while *IbSAP5* was rapidly induced after 1 h of salinity treatment. The expression of *IbSAP*s increased up to 20-fold (*IbSAP16* at 12 h salinity treatment) compared to control conditions. The mRNA levels of two *IbSAP*s (*IbSAP18* and *IbSAP20*) under salinity treatment, five *IbSAP*s (*IbSAP5*, *16*, and *18–20*) under drought treatment, and three *IbSAP*s (*IbSAP3*, *5*, and *20*) under ABA treatment kept increasing during the 24-h treatment. These results indicated that almost all of the *IbSAP*s can be induced by salinity, drought, or ABA treatment.

**Figure 7:**
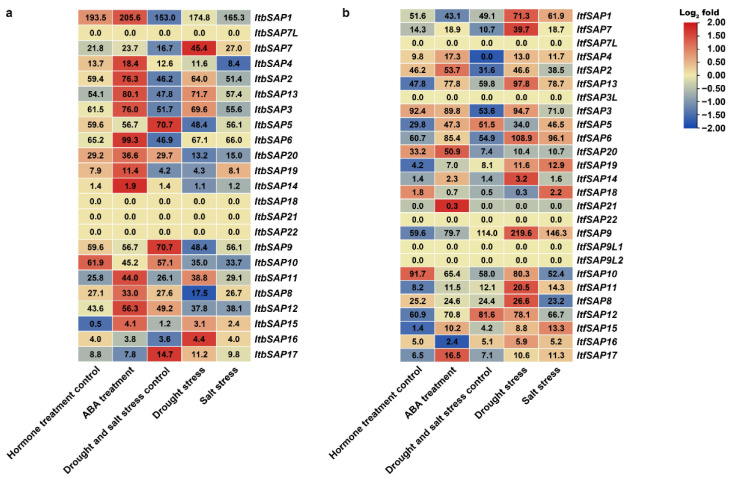
Gene expression patterns of *ItbSAP*s (**a**) and *ItfSAP*s (**b**) in response to ABA treatment, drought stress, or salinity stress as determined by RNA-seq. FPKM data are shown in the boxes.

In addition, the expression patterns of *I. triloba* and *I. trifida SAPs* in response to ABA, salinity, and drought treatments were also analyzed using the RNA-seq data [30]. Under ABA treatment, 14 *ItbSAPs* and 16 *ItfSAPs* were induced, while 5 *ItbSAPs* and 6 *ItfSAPs* were repressed. Under salinity treatment, 10 *ItbSAPs* and 17 *ItfSAPs* were induced, while 9 *ItbSAPs* and 3 *ItfSAPs* were repressed. Under drought treatment, 10 *ItbSAPs* and 16 *ItfSAPs* were induced, while 9 *ItbSAPs* and 4 *ItfSAPs* were repressed. Four *ItbSAPs* and six *ItfSAPs* were not detected under those treatments. These results indicated that most *ItbSAPs* and *ItfSAPs* respond to salinity, drought, or ABA treatments, and they might play important roles in abiotic stress responses in *I. triloba* and *I. trifida*. Expression patterns of *SAP* orthologs in *I. triloba* and *I. trifida* were similar (Figure 7). However, most *IbSAPs* were induced by stress treatments and no *IbSAPs* were repressed (Figure 6).

### 2.7. Ectopic Expression of IbSAP16 Enhanced Salinity Tolerance of Transgenic Arabidopsis Plants

Many *SAP* genes regulate stress tolerance in plants [3,7,8,9,10,11,12]. However, the mechanism of SAP proteins containing only AN1 domains or AN1 and C_2_H_2_ domains in the stress response is not well-studied. IbSAP16 is one of the two AN1-AN1-C_2_H_2_-C_2_H_2_ type SAPs in sweetpotato, and the other one is IbSAP17 (Figure 3). *IbSAP16* was upregulated more than 20-fold after salinity stress, however the expression level of *IbSAP17* changed less than 2-fold (Figure 6). Therefore, to test the hypothesis that *IbSAP16* confers salinity tolerance, we ectopically expressed *IbSAP16* driven by the 35 S promoter in *Arabidopsis thaliana Col*-0 (WT). Two independent *IbSAP16*-overexpressing transgenic *Arabidopsis* lines (L5 and L22) with different *IbSAP16* mRNA levels (Figure S3) were selected for further study.

**Figure 8:**
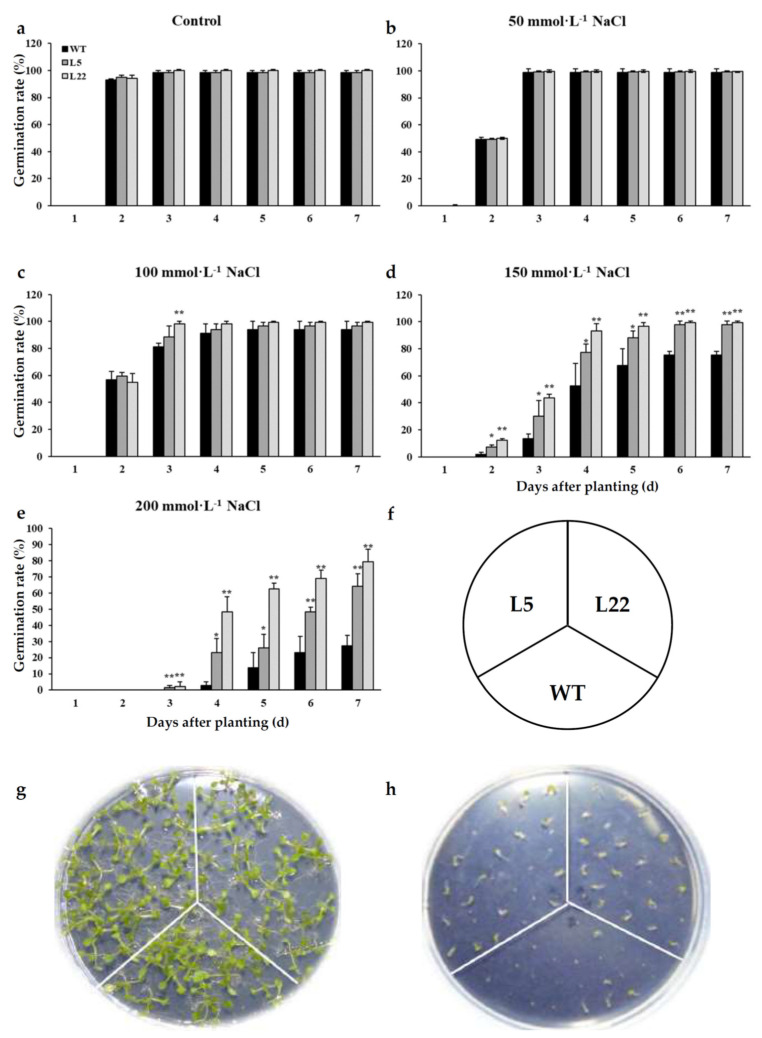
Germination of *IbSAP16*-transgenic *Arabidopsis* in response to NaCl treatment. Seeds of WT and *IbSAP16*-transgenic *Arabidopsis* lines were plated on 1/2 Murashige and Skoog (MS) medium with different concentrations of NaCl. Germination rates on 1/2 MS (**a**), 1/2 MS + 50 mmol·L^−1^ NaCl (**b**), 1/2 MS + 100 mmol·L^−1^ NaCl (**c**), 1/2 MS + 150 mmol·L^−1^ NaCl (**d**), and 1/2 MS + 200 mmol·L^−1^ NaCl (**e**) were measured. (**f**) Seeding diagram; (**g**) germination on control media; (**h**) germination on media with 200 mmol·L^−1^ NaCl. Error bars represent standard error (SE) based on three independent replicates. “*” or “**” indicate a significant difference from that of WT at *p* < 0.05 or 0.01, by Student’s *t*-test.

To investigate the function of *IbSAP16* in the salinity stress response, germination rates of L5, L22, and WT under salinity stress were investigated (Figure 8). Without NaCl treatment, or under 50 and 100 mmol·L^−1^ NaCl treatment, germination rates of *IbSAP16-*transgenic lines were similar to WT. Only on day 3 on media with 100 mmol·L^−1^ NaCl, the germination rate of L22 was significantly higher than that of WT. Finally, approximately 100% of L5, L22, and WT seeds germinated on the 1/2 Murashige and Skoog (MS) media with 0, 50, or 100 mmol·L^−1^ NaCl. When NaCl concentration was increased to 150 or 200 mmol·L^−1^, germination rates of L5 and L22 seeds were significantly higher than those of WT seeds (Figure 8a–e). These results suggested that ectopic expression of *IbSAP16* enhanced salinity tolerance of transgenic *Arabidopsis* at the germinating stage.

**Figure 9:**
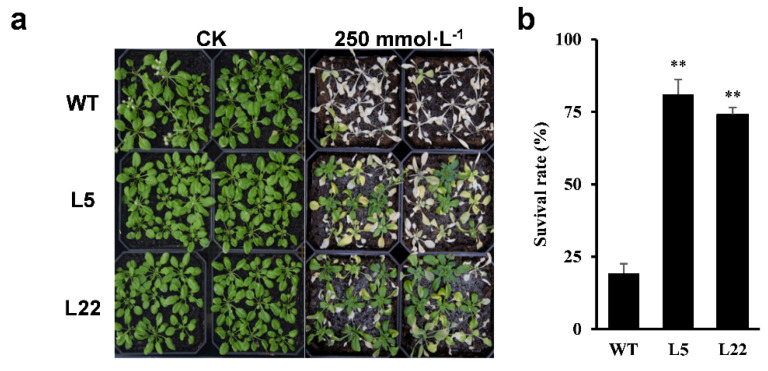
Ectopic expression of *IbSAP16* enhanced salinity tolerance in transgenic *Arabidopsis*. Four-week-old WT and transgenic *Arabidopsis* seedlings were treated with 250 mmol·L^−1^ NaCl solution for 3 weeks. (**a**) Representative image. (**b**) Survival rates of WT and transgenic lines after NaCl treatment. Data are presented as mean ± SE (n = 3). “**” indicates a significant difference from that of WT at *p* < 0.01, by Student’s *t*-test.

Furthermore, survival rates of L5, L22, and WT seedlings under salinity stress were determined. While all seedlings survived under control conditions, only 19.3% of WT seedlings survived under 250 mmol·L^−1^ NaCl treatment, but 81.0% and 74.1% of L5 and L22 seedlings, respectively, survived the 250 mmol·L^−1^ NaCl treatment (Figure 9). These results suggested that ectopic expression of *IbSAP16* enhanced salinity stress tolerance of transgenic *Arabidopsis* at the seedlings stage.

## 3. Discussion

A20/AN1 zinc finger domain containing proteins play a pivotal role in regulating immune responses in animals and abiotic stress responses in plants. They have been widely studied for their participation in physiological and biochemical reactions such as immune responses and apoptosis. Human ZNF216, containing both A20 and AN1 domains, regulates immune responses by inhibiting the activation of nuclear factor-kappa β (NF-κB). Consequently, ZNF216 reduces allergic reactions and cell apoptosis [31,32]. Protein A20 inhibits the activity of NF-κB as well, which reduces cell apoptosis caused by the tumor necrosis factor (TNF) [4,33,34]. Investigations of SAP proteins in plants were conducted much later than those in animals. The first A20/AN1 domain containing protein encoding gene identified in plants was *OsiSAP1*, which is induced by multiple abiotic stresses. Ectopic expression of *OsiSAP1* in tobacco conferred tolerance to abiotic stresses [3]. Up to now, *SAPs* in rice, *Arabidopsis*, maize, polar, tomato, cotton, *Medicago truncatula*, apple, *Brassica napus*, soybean, and cucumber were identified [2,6,17,18,19,20,21,22,23]. However, the function of the *SAP* family in sweetpotato is still poorly understood.

### 3.1. Evolution of the SAP Gene Family in Sweetpotato and Its Two Diploid Relatives

In this study, a total of 69 *SAPs* were identified in hexaploid sweetpotato and its two diploid relatives, including 20 *IbSAPs*, 23 *ItbSAPs*, and 26 *ItfSAPs* (Table 1 and Table S1, and Figure 1), which were divided into four clades (clade I to IV) according to their structures (Figure 2). The number of *IbSAP* genes (20) is more than that found in diploid plants, such as *A. thaliana* (14), rice (18), maize (11), and tomato (13) [2,6,17]. However, in some polyploidy crops, the number of A20/AN1 zinc finger encoding genes is more than that in sweetpotato. For example, 37 and 57 *SAP* genes were identified in tetraploid crops, cotton [18] and *B. napus* [21], respectively. In addition, in two diploid wild relatives of sweetpotato, more SAP genes were identified. No orthologs of two *ItbSAP*s (*ItbSAP21* and *ItbSAP22*) and two *ItfSAP*s (*ItfSAP21* and *ItfSAP22*) were identified in the sweetpotato. Sweetpotato is a hexaploid crop (2x = 6n = 90); however, the genome sequence was assembled into only 15 pairs rather than 45 pairs of chromosomes [29]. Thus, there should additional *IbSAP* gene members or gene copies in the sweetpotato genome. Moreover, only 18 of 20 *IbSAPs* were identified from the sweetpotato genome database [29], while *IbSAP13* and *IbSAP17* were identified from our unpublished transcriptome database. One of the reasons of this result might be that genome sequences vary between different sweetpotato varieties, and some new *SAPs* emerged after sweetpotato evolved from the ancestor species. The other reason might be that some *IbSAPs* are located in chromosomes with relatively poor sequencing quality. Nevertheless, our results supplement the sweetpotato genome database, but further improvement of the sweetpotato genome sequence is urgently needed to promote research on sweetpotato gene functions.

The gene structures of most orthologous *SAPs* were similar between sweetpotato and its wild relatives—*I. triloba* and *I. trifida*—except that *IbSAP1*, *6*, *7*, and *12* were different from their orthologs in *I. triloba* and *I. trifida*. The differences were mainly intron insertion or deletion. Meanwhile, the gene structures of these orthologous SAPs in *ItbSAPs* and *ItfSAPs* were almost the same (Figure 3b). This result suggested that the *SAP* gene family is relatively conserved, but still some changes occurred during or after formation of the hexaploid species from its diploid relatives.

Many SAPs in plants contain a typical SAP structure (an AN1 domain at the C-terminus and an A20 domain at the N-terminus) [1]. Ten out of 14 SAP proteins in *Arabidopsis*, 11 out of 18 in rice, 9 out of 13 in tomato, and 8 out of 11 in maize are typical SAPs [2,6,17]. Similarly, we found that 14 out of 20 IbSAPs, 14 out of 23 ItbSAPs, and 15 out of 26 ItfSAPs contain the typical SAP structure (Figure 3c and Figure S1). The other Ib/Itb/ItfSAP proteins contain 1 or 2 AN1 domains with 0 or 2 C_2_H_2_ zinc finger domains, but no A20 domains (Ib/Itb/ItfSAP6 were exceptions, which contain one incomplete A20 domain). We found that the AN1 domain in the five IbSAPs lacking A20 domains was CX_4_CX_9-12_CX_1-2_CX_4_CX_2_HX_5_HXC, which is different from the typical AN1 (CX_2_CX_9-12_CX_1-2_CX_4_CX_2_HX_5_HXC) in those SAPs harboring A20 domains. There is a two-amino-acid addition at the N-terminus in the former AN1, which is classified as an expanded AN1 [6]. This observation was in agreement with previously published results [6]. However, we identified a few differences. All “Type I” SAP proteins (clades I, II, and III in this study) other than SlSAP10 had the typical AN1 domain, while “Type II” SAPs (clade IV in this study) had the expanded AN1 domain. The A20-lacking SAPs, SAP15, SAP16, and SAP17 of sweetpotato, *I. triloba*, and *I. trifida* belong to clade IV, while SAP8 and SAP12 belong to clade III. However, SAP8 and SAP12 of these three *Ipomoea* plants and their ortholog in tomato (SlSAP10) had the expanded pattern of the AN1 domain. In addition, only Ib/Itb/ItfSAP6 in clade II had an incomplete A20 domain (Figure 3 and Figure S1). These results suggest that special evolutionary events have occurred in *SAP* genes in *Ipomoea* or Solanales species.

To date, the evolutionary history of the sweetpotato is still debated. It is, however, commonly accepted that *I. triloba* and *I. trifida* are closely related to sweetpotato [35,36]. In this study, we identified *IbSAP*s in sweetpotato and their orthologs in *I. triloba* and *I. trifida*. These three *SAP* gene families share high similarities, which provided further evidence for the theory that *I. triloba* and *I. trifida* might be the ancestral species of sweetpotato.

### 3.2. Different Functions of SAPs on Growth and Development between Sweetpotato and Its Two Diploid Relatives

SAPs play pivotal roles in growth and development of plants. *OsSAP4* in rice is constitutively expressed and negatively regulates the synthesis of gibberellin, thus controlling plant height, leaf blade size, and grain size [37]. *AtSAP5* is expressed throughout *A. thaliana* plants and positively regulates plant growth by mediating the degradation of AtMBP-1 [38]. Similarly, most of the *IbSAPs*, *ItbSAPs*, and *ItfSAPs* were expressed in all organs tested. Meanwhile, mRNA levels of *SAP*s varied greatly among different organs of sweetpotato, as well as in its two diploid relatives (Figure 4 and Figure 5). The expression level of *IbSAP10* in pencil roots was 100-fold higher than the *IbSAP18* level in tuberous roots. Similar results were previously reported for expression patterns of tomato *SAPs*, for instance, the mRNA level of *SlSAP1* was approximately 120-fold higher than *SlSAP13* mRNA levels in unstressed 9-day-old tomato seedlings [17].

Most *IbSAPs* shared similar expression patterns in different organs with their orthologs in *I. triloba* and *I. trifida*. However, there were some exceptions, and these distinguished expression patterns of *SAP* orthologs in *I. batatas*, *I. triloba*, and *I. trifida* might indicate different functions. *IbSAP16* in sweetpotato might mainly regulate leaf development, while its orthologs in *I. triloba* and *I. trifida* might mainly regulate root development. This is because *IbSAP16* has higher expression levels in leaves than in other organs (Figure 4), while *ItbSAP16* and *ItfSAP16* has higher expression levels in roots (Figure 5). Moreover, *IbSAP3* might play an important role in the development of tuberous roots in sweetpotato, because it is highly expressed in tuberous roots (Figure 4). *I. triloba* and *I. trifida* do not have tuberous roots, while *ItbSAP3* and *ItfSAP3* has higher expression levels in stems and leaves than in root (Figure 5). These results suggest that most orthologs of *SAPs* in *I. triloba* and *I. trifida* might play similar roles in growth and development, but that they have derived new functions when the two ancestors evolved into sweetpotato.

### 3.3. Different Functions of SAPs in Multiple Abiotic Stress Responses between Sweetpotato and Its Two Diploid Relatives

Many *SAP* genes are induced by various abiotic stresses, and overexpression or ectopic expression of certain *SAP* genes confers stress tolerance in transgenic plants. For example, the first *SAP* gene identified in plants, *OsiSAP1*, is induced by abiotic stresses such as salinity, desiccation, submergence, heavy metals, mechanical wounding, and ABA treatment [3]. Ectopic expression or overexpression of *OsiSAP1* confers tolerance to abiotic stress in transgenic tobacco [3], *Arabidopsis* [7], and rice [8]. An *Arabidopsis SAP* gene, *AtSAP12*, is responsive to cold, drought, genotoxic, osmotic, oxidative, salinity, and wounding stresses [39]. Similar to *SAP*s in other plants, *IbSAP*s are induced by salinity, dehydration, or ABA treatment (Figure 6), which most likely is due to the presence of stress response and ABA response *cis*-acting elements in promoter regions of *IbSAP*s (Table 2, Figure S2). Many *SAPs* from other plants have been reported to be induced rapidly by multiple abiotic stresses. OsSA*P1, AtSAP10*, and *MusaSAP1* (*SAP1* in banana) are induced within 15 or 30 min after exposure to various stresses [3,40,41]. We found that some *IbSAP*s could also rapidly respond to abiotic stresses. *IbSAP1*, *IbSAP3*, *IbSAP4*, and *IbSAP5* are upregulated just 1 h after salinity, drought, or ABA treatments, and *IbSAP5* even had the highest mRNA levels just 1 h after salinity treatment (Figure 6). Most *IbSAP*s reached their peak value 6 h after stress treatments. By contrast, other *IbSAP*s reached their highest mRNA levels 12 h after treatments, such as *IbSAP14* and *IbSAP16* in response to salinity and ABA treatments, and *IbSAP8* in response to salinity and drought treatments. These results indicated that *IbSAPs* might function at different stages of stress responses in sweetpotato.

Some A20-lacking *SAPs* have functional redundancy in response to abiotic stresses. Compared to wild type, homozygous loss-of-function *Arabidopsis* mutants of *SAP* genes encoding two AN1 domains have no visible phenotypic differences under various stress conditions [6]. In our research, *IbSAP1* encoding a protein containing two AN1 and two C_2_H_2_ domains, seemed to be insensitive to abiotic stresses. The expression level of *IbSAP17* changed less than 2-fold after salinity, drought, and ABA treatments (Figure 6). This result suggested functional redundancy of A20-lacking *SAPs* in response to abiotic stresses as well. However, further investigations are needed to confirm this assumption.

In addition, hormone response *cis*-acting elements for auxin, ethylene, gibberellin, salicylic acid, and methyl jasmonate, and light signal response *cis*-acting elements were found in promoter regions of *IbSAPs* (Table 2 and Figure S2). Some *SAPs* were previously reported to be regulators of biotic stress responses or plant growth and development, such as *OsDOG* (*OsSAP11*), which is induced by gibberellins and negatively regulates rice cell elongation [42]. *AtSAP9*-overexpressing plants showed delayed flowering time and were more susceptible to *P. syringae* pv. *phaseolicola* infections [13]. Moreover, *AtSAP5*, *Pha13*, and *SlSAP3* are involved in regulating resistance to viral and bacterial infections [15,16]. *IbSAP*s might be involved in biotic stress responses and regulation of plant growth and development as well. This is worth investigating, because sweetpotato virus disease (SPVD) has been seriously affecting the production of sweetpotato [43].

In addition, expression patterns of orthologous *SAPs* in diploid *I. triloba* and *I. trifida* were different from those in sweetpotato (Figure 7). Some *Itb/ItfSAPs* were induced by salinity and drought stresses and ABA treatment as well; however, some *Itb/ItfSAPs* were repressed. These stress-induced or different expression patterns of *Ib/Itb/ItfSAPs* homologs in response to abiotic stress could both provide potential candidate genes for further functional research and for modifying abiotic stress tolerance of plants.

### 3.4. AN1-AN1-C_2_H_2_-C_2_H_2_ Type SAPs Can Be Candidates to Improve Salinity Stress Tolerance of Plants

Most functional analyses of SAPs in plants have focused on typical SAP proteins, which have one AN1 domain and one A20 domain, while only a few studies have investigated SAPs containing only AN1 domains [22,39,44,45] and AN1-AN1-C_2_H_2_-C_2_H_2_ type SAPs [11]. *IbSAP16* is one of the two AN1-AN1-C_2_H_2_-C_2_H_2_ encoding genes in sweetpotato. Its expression was induced by salinity treatment. The ortholog of *IbSAP16* in *A. thaliana*—*AtSAP13*—positively regulates tolerance to multiple abiotic stresses [11]. In our study, we found that *IbSAP16-*transgenic *Arabidopsis* lines showed stronger tolerance to salinity stress in both germination and vegetative growth stages (Figure 8 and Figure 9). These results indicated that *IbSAP16* from sweetpotato also functions as a positive regulator in response to abiotic stresses, and AN1-AN1-C_2_H_2_-C_2_H_2_ type *SAPs* should be considered as candidates for improving salinity stress tolerance in plants. These results also laid the foundation for IbSAP16-mediated abiotic stress response mechanism studies.

## 4. Materials and Methods

### 4.1. Characterization of SAP Members in Sweetpotato and Its Two Relative Species

The hidden Markov models of A20 zinc finger (pfam01754) and AN1 zinc finger (pfam01428) obtained from EMBL-EBI PFAM (http://pfam.xfam.org/, accessed on 2 December 2019) [46] were used in a query for scanning against sweetpotato protein database downloaded from *Ipomoea* Genome Hub (https://www.ipomoea-genome.org, accessed on 2 December 2019) [29] and our transcriptome data of sweetpotato with HMMER v3.1. The sequences obtained by HMMER were then analyzed with the NCBI branch web CD-search tool (www.ncbi.nlm.nih.gov/Structure/bwrpsb/bwrpsb.cgi, accessed on 2 December 2019) [47] and PFAM (http://pfam.xfam.org/search, accessed on 2 December 2019) [46], and sequences without A20/AN1 domains or with incomplete A20/AN1 were eliminated. All remaining protein sequences were recognized as putative *Ipomoea batatas* stress-associated proteins (IbSAPs). In addition, SAP members in two diploid wild relatives of sweetpotato, *Ipomoea triloba* and *Ipomoea trifida*, were searched with the same method against *I. triloba* and *I. trifida* genome database in Sweetpotato Genomics Resource (http://sweetpotato.plantbiology.msu.edu/index.shtml, accessed on 2 December 2019) [30]. The theoretical isoelectric points (pI), molecular weight (Mw) and grand average of hydropathicity (GRAVY) of IbSAPs were computed using the ExPASy ProtParam tool (https://web.expasy.org/protparam/, accessed on 2 December 2019) [48], and the subcellular localization was predicted by LOCTREE 3 (https://rostlab.org/services/loctree3/, accessed on 3 December 2019) [49].

### 4.2. Chromosomal Distribution of IbSAPs, ItbSAPs, and ItfSAPs

The location information of *IbSAPs* were obtained from the sweetpotato gff3 annotation file downloaded from the *Ipomoea* Genome Hub (https://sweetpotao.com/, accessed on 2 December 2019). The location information of *ItbSAPs* and *ItfSAPs* were obtained from gff3 annotation downloaded from Sweetpotato Genomics Resource (http://sweetpotato.plantbiology.msu.edu/, accessed on 2 December 2019). The *SAP* genes in *I. batatas*, *I. triloba*, and *I. trifida* with location information were mapped to chromosomes by TBtools software [50].

### 4.3. Phylogenetic Analysis of IbSAPs with SAPs from Six Other Plants

The amino acid sequences of AtSAPs, OsSAPs, SlSAPs, and ZmAN1s were downloaded from TAIR (http://www.Arabidopsis.org/, accessed on 2 December 2019), PlantGDB (http://www.plantgdb.org/, accessed on 2 December 2019), or Phytozome (https://phytozome.jgi.doe.gov/pz/portal.html, accessed on 2 December 2019) [51] using previously published accession numbers [2,6,17]. The SAP protein sequences of *Ipomoea batatas*, *Ipomoea triloba, Ipomoea trifida*, *Arabidopsis thaliana, Oryza sativa, Solanum lycopersicum,* and *Zea mays* were then aligned by MEGA-X [52] using the MUSCLE method. An unrooted phylogenetic tree of IbSAPs, ItbSAPs, ItfSAPs AtSAPs, OsSAPs, SlSAPs, and ZmAN1s was constructed using the neighbor-joining method with the alignment results. The phylogenetic tree was visualized by EvolView (www.evolgenius.info/evolview/, accessed on 13 April 2022) [53]. An unrooted phylogenetic tree of IbSAPs was also constructed by the same method.

### 4.4. Analyses of Gene Structure and Conserved Domains of SAP Members

Gene structure information was obtained from gff3 annotation of *I. batatas*, *I. triloba*, and *I. trifida*. Conserved domains were analyzed by the NCBI branch web CD-search tool as mentioned above. Gene structures and conserved domains were generated using TBtools software [50].

### 4.5. Cis-Acting Regulatory Elements Analyses of IbSAPs

For promoter analyses, genomic DNA sequences 2 kb upstream of the translation initiation site of *IbSAPs* were extracted from the sweetpotato genome database. *Cis*-acting regulatory elements in the upstream sequences were then analyzed using the online database PlantCARE (http://bioinformatics.psb.ugent.be/webtools/plantcare/html/, accessed on 26 May 2020) [54].

### 4.6. Plant Materials

Sweetpotato (*Ipomoea batatas*) cultivar Xushu 28, a variety developed in our institute with high yield and salinity tolerance, was used in this study, and the sampling permission was obtained. Leaf, stem, tuberous root, pencil root, and fibrous root were sampled 90 d after planting for quantitative real-time PCR analyses of *IbSAP*s in different organs. Shoots of 20 cm length were cultured in a one-quarter Hoagland solution and kept in an MLR-352 Panasonic illumination incubator at 25 ± 1 °C with 16 h light/8 h dark cycles. The light intensity of the illumination incubator was set at LS5 (21637 lx). *Arabidopsis* plants were kept in a growth chamber (22 °C, 16 h light/8 h dark cycle) for functional analysis of *IbSAP16*.

### 4.7. Stress Treatments of Sweetpotato Shoots

Sweetpotato shoots were treated with 100 mmol·L^−1^ NaCl (salinity stress), 15% polyethylene glycol (PEG) 6000 (simulated drought stress), or 10 μmol·L^−1^ ABA in one-quarter Hoagland solution after their roots grew to 3–5 cm. Shoots treated with one-quarter Hoagland solution was used as control (CK). The roots were sampled at 0, 1, 6, 12, and 24 h after treatments for quantitative real-time PCR analysis of *IbSAP*s. All samples were frozen in liquid nitrogen right away and stored at −70 °C before RNA isolation.

### 4.8. RNA Isolation and Quantitative Real-Time PCR Analysis

RNA was isolated using the Generay total RNA isolation kit (Shanghai Generay Biotech Co., Ltd., Shanghai, China, code no. GK3016) and treated with DNase I following the instructions of the manufacturer. RNA samples were checked by denaturing agarose gel electrophoresis and ND-1000 to determine RNA quality and concentration. First strand cDNA was synthesized from 2 μg total RNA using the ReverTra Ace qPCR RT Master Mix with gDNA Remover (Toyobo, Japan, code no. FSQ-301). The primers used for RT-qPCR were designed using Primer 3 Plus (http://www.bioinformatics.nl/cgi-bin/primer3plus/primer3plus.cgi, accessed on 3 December 2019) [55] and were listed in Table S3. Real-time qPCR was performed using the Toyobo SYBR Green Realtime PCR Master Mix (TOYOBO Co., Ltd. Japan, code no. QPK-201). The reaction cycles were set as follows: 95 °C (2 min)/(95 °C (15 s)/60 °C (15 s)/72 °C (20 s) × 40 cycles); the melt curve of each reaction was detected per 0.3 °C from 60 °C to 95 °C. These reactions were put in 96-well optical reaction plates (Applied Biosystems, Foster City, CA, USA) and carried out in ABI StepOne Plus real-time PCR system. Each reaction was repeated three times. *IbARF* (*Ipomoea batatas ADP-ribosylation factor*, GenBank accession no. JX177359) and *AtActin2* were used as the internal control to normalize the variation among sweetpotato [56] and *Arabidopsis* templates, respectively. The relative mRNA levels were calculated using the 2^−ΔΔCt^ method [57].

### 4.9. Transcriptome Analysis of ItbSAPs and ItfSAPs Expression Patterns

The RNA-seq data of *I. triloba* and *I. trifida* were obtained from the Sweetpotato Genomics Resource (http://sweetpotato.plantbiology.msu.edu/, accessed on 2 December 2019) [30]. The value of fragments per kilobase of exon per million fragments mapped (FPKM) were used to represent the relative expression level of *ItbSAPs* and *ItfSAPs*. The heat maps were visualized by TBtools software [50].

### 4.10. Plant Expression Vector Construction of IbSAP16 and Arabidopsis Transformation

Full-length CDS of *IbSAP16* was cloned from cDNA of Xushu 28 using primers shown in Supplementary Table S1 and ligated into vector pCR8/GW/TOPO (Invitrogen, Carlsbad, CA, USA, code no. K252020) to obtain pCR8/GW/TOPO-*IbSAP16*. pGWB12-*IbSAP16* (*35S::IbSAP16*) was constructed by LR reaction between pGWB12 and pCR8/GW/TOPO-*IbSAP16*. *Agrobacterium tumefaciens* strain GV3101 was used to transfer *35S::IbSAP16* into *Arabidopsis* by using the floral dipping method [58]. T1 generation *Arabidopsis* lines were selected by germinating the seeds on half MS media with 50 mg·L^−1^ hygromycin and confirmed by PCR and RT-qPCR. T3 homozygous lines were screened and used for further experiments.

### 4.11. Assay for Salinity Tolerance of Transgenic Arabidopsis

For germination-rate assays, sterilized *Arabidopsis* seeds from *IbSAP16*-transgenic lines and WT plants were planted on 1/2 MS media with 0, 50, 100, 150, and 200 mmol·L^−1^ NaCl and kept at 4 °C for 2 days before they were cultured in a growth chamber (22 °C, 16 h light/8 h dark cycle). Germination rates were recorded daily within a week. Seven-day-old seedlings with similar growth rates were transplanted to pots. Three weeks later, the plants were watered with 250 mM NaCl solution or pure water (300 mL for each pot) once a week for 3 weeks to determine their survival rates under salinity stress.

### 4.12. Statistical Analysis

SPSS 22 Statistical software was used in statistical analysis. Means and standard errors were calculated and analyzed with ANOVA, and the probabilities for significance were estimated with Student’s *t*-test.

## 5. Conclusions

In this study, 20, 23, and 26 *SAP* genes encoding A20/AN1 zinc finger proteins were identified in *I. batatas*, *I. triloba*, and *I. trifida*. Two of the 20 *IbSAPs* were only identified in our unpublished transcriptomics database, which supplements the sweetpotato genome database. The gene structures and conserved protein domains of *SAP* family members in sweetpotato and their orthologs in *I. triloba* and *I. batatas* were similar. Sequences alignment of SAP in sweetpotato, *I. triloba*, *I. trifida*, and other plants revealed Solanales-specific AN1 zinc finger domain in SAPs containing only one AN1 zinc finger domain, which indicated specific evolution events occurred in *SAP* gene families of Solanales plants. The different organ-specific and stress response expression patterns of *IbSAPs*, *ItbSAPs*, and *ItfSAPs* suggested that these *SAP* genes are involved in plant growth and development, as well as abiotic stress responses. In addition, we showed that *IbSAP16*—an AN1-AN1-C_2_H_2_-C_2_H_2_ type *IbSAP—*is a positive regulator in response to salinity stress in transgenic *A. thaliana*. These results provided a characterization of *SAP* genes in *I. batatas*, *I. triloba*, and *I. trifida* and laid the groundwork for studying IbSAP-mediated stress response mechanisms. The function and regulatory network of certain *SAP*s in sweetpotato, *I. triloba*, and *I. trifida* merits further investigation.

## Figures and Tables

**Table 1:** A20/AN1 zinc finger domain containing proteins in sweetpotato.

Gene ID	Locus ^1^	CDS Length (bp)	Protein Length (aa)	pI	Mw (kDa)	GRAVY	Predicted Subcellular Localization
*IbSAP1*	g30531	507	168	7.98	17.87	−0.504	cytoplasm
*IbSAP2*	g43065	525	174	7.98	18.32	−0.346	cytoplasm
*IbSAP3*	g11483	516	171	7.51	18.28	−0.488	cytoplasm
*IbSAP4*	g57575, g57655	507	168	7.49	18.11	−0.388	cytoplasm
*IbSAP5*	g50881	513	170	7.49	18.07	−0.379	cytoplasm
*IbSAP6*	g58165	456	151	9.05	16.32	−0.418	cytoplasm
*IbSAP7*	g8952	489	162	8.52	17.29	−0.430	cytoplasm
*IbSAP8*	g50882	429	142	8.67	15.59	−0.601	cytoplasm
*IbSAP9*	g30530	519	172	9.06	19.12	−0.935	cytoplasm
*IbSAP10*	g8953	525	174	9.14	19.07	−0.726	cytoplasm
*IbSAP11*	g57577, g57652	501	166	9.36	18.18	−0.713	cytoplasm
*IbSAP12*	g4341	507	168	8.72	18.20	−0.813	cytoplasm
*IbSAP13*	/	537	178	8.81	18.63	−0.365	cytoplasm
*IbSAP14*	g38216	474	157	9.48	16.89	−0.492	cytoplasm
*IbSAP15*	g30138	579	192	9.06	20.96	−0.588	endoplasmic reticulum
*IbSAP16*	g43042	834	277	8.63	30.59	−0.577	endoplasmic reticulum
*IbSAP17*	/	885	294	8.63	32.56	−0.640	nucleus
*IbSAP18*	g15701, g19161	465	154	8.65	16.71	−0.501	cytoplasm
*IbSAP19*	g25751	528	175	8.79	18.48	−0.608	cytoplasm
*IbSAP20*	g25129	435	144	9.21	15.40	−0.363	cytoplasm

^1^ Locus IDs were obtained from the *Ipomoea* Genome Hub (https://www.ipomoea-genome.org, accessed on 2 December 2019).

**Table 2:** *Cis*-acting elements in 2 kb promoter region of *IbSAP*s

*Cis*-Acting Elements	Functions	Sequences	Genes
Hormone response cis-acting elements			
ABRE	*cis*-acting element involved in the abscisic acid responsiveness	ACGTG	*IbSAP2–5, 8–12, 14–16, 19*
as-1	involved in the response to auxin, salicylic acid, and methyl jasmonate	TGACG	*IbSAP1, 3–5, 7–12, 14, 15, 18, 19*
ERE	ethene responsive element	ATTTCATA/ ATTTTAAA	*IbSAP2, 5, 6, 8–12, 15, 16, 18–20*
GARE-motif	gibberellin-responsive element	TCTGTTG	*IbSAP1, 7, 8, 12, 16*
P-box	gibberellin-responsive element	CCTTTTG	*IbSAP1, 2, 4, 11, 12, 14, 15, 20*
TCA-element	*cis*-acting element involved in salicylic acid responsiveness	TCATCTTCAT/ CCATCTTTTT/ TCAGAAGAGG	*IbSAP3, 4, 5, 7, 8, 10, 15, 16, 19*
TGA-box	auxin-responsive element	TGACGTAA/ AACGAC	*IbSAP1, 7–11, 14, 16, 19*
Stress response cis-acting elements			
DRE	drought responsive element	GCCGAC/ ACCGAGA	*IbSAP3, 5, 8, 15*
LTR	*cis*-acting element involved in low-temperature responsiveness	CCGAAA	*IbSAP2, 3, 5, 7, 8, 11, 15, 18, 19*
MBS	MYB binding site involved in drought-inducibility	CAACTG	*IbSAP2, 7, 8–10, 12, 15, 16*
MYB	MYB binding site	CAACGG/CAACAG/ CAACCA/TAACCA/ TAACTG/CAACTG/ CCGTTG	*IbSAP1-IbSAP12, 14–16, 18–20*
STRE	stress-responsive elements	AGGGG	*IbSAP2, 3, 5–11, 15, 16, 18, 20*
TC-rich repeats	*cis*-acting element involved in defense and stress responsiveness	ATTCTCTAAC	*IbSAP1, 2, 5, 8*
W-box	*cis*-acting element involved in sugar metabolism and plant defense signaling	TTGACC	*IbSAP1, 2, 4–6, 9, 10, 12, 14–16, 18, 20*
WRE3	wound-responsive element	CCACCT	*IbSAP2–5, 7, 11, 15, 18–20*
WUN-motif	wound-responsive element	AAATTACT/ AAATTTCTT/ TTATTACAT/ CAATTACAT/ AAATTTCCT	*IbSAP1, 5, 6, 8, 10, 12, 14, 16, 18–20*
Light signal response cis-acting elements			
Box 4	part of a conserved DNA module involved in light responsiveness	ATTAAT	*IbSAP2–6, 8–12, 14, 15, 18–20*
TCT-motif	part of a light responsive element	TCTTAC	*IbSAP3, 5–8, 11, 14, 15, 18, 19*
Sp1	light responsive element	GGGCGG	*IbSAP3, 9, 11, 12*
chs-CMA1a/2a	part of a light responsive element	TTACTTAA/ TCACTTGA	*IbSAP3–10*
GATA-motif	part of a light responsive element	AAGATAAGATT/ AAGGATAAGG/ GATAGGA/ GATAGGG	*IbSAP1, 4, 6, 8–11, 14–16, 18, 20*
G-box	*cis*-acting regulatory element involved in light responsiveness	TACGTG/ CACGTG/CACGTC /CACGTT/CACGAC	*IbSAP2–12, 14–16, 19*
GT1-motif	light responsive element	GGTTAA	*IbSAP3, 6, 7, 9, 12, 14, 19, 20*

## Data Availability

The data presented in this study are available on request from the corresponding author.

## Supplementary Materials

The following supporting information can be downloaded at: https://www.mdpi.com/article/10.3390/ijms231911551/s1.
