# Supplementary material for: Genome-Wide Identification of the A20/AN1 Zinc Finger Protein Family Genes in Ipomoea batatas and Its Two Relatives and Function Analysis of IbSAP16 in Salinity Tolerance
Source: Int J Mol Sci. 2022 Sep 30;23(19):11551. doi: 10.3390/ijms231911551 (PMC9570247; doi:10.3390/ijms231911551)
Supplement: Supplementary file 1 [file ijms-23-11551-s001.zip › supplementary figure 20220926.pdf]

Supplementary Figures

a

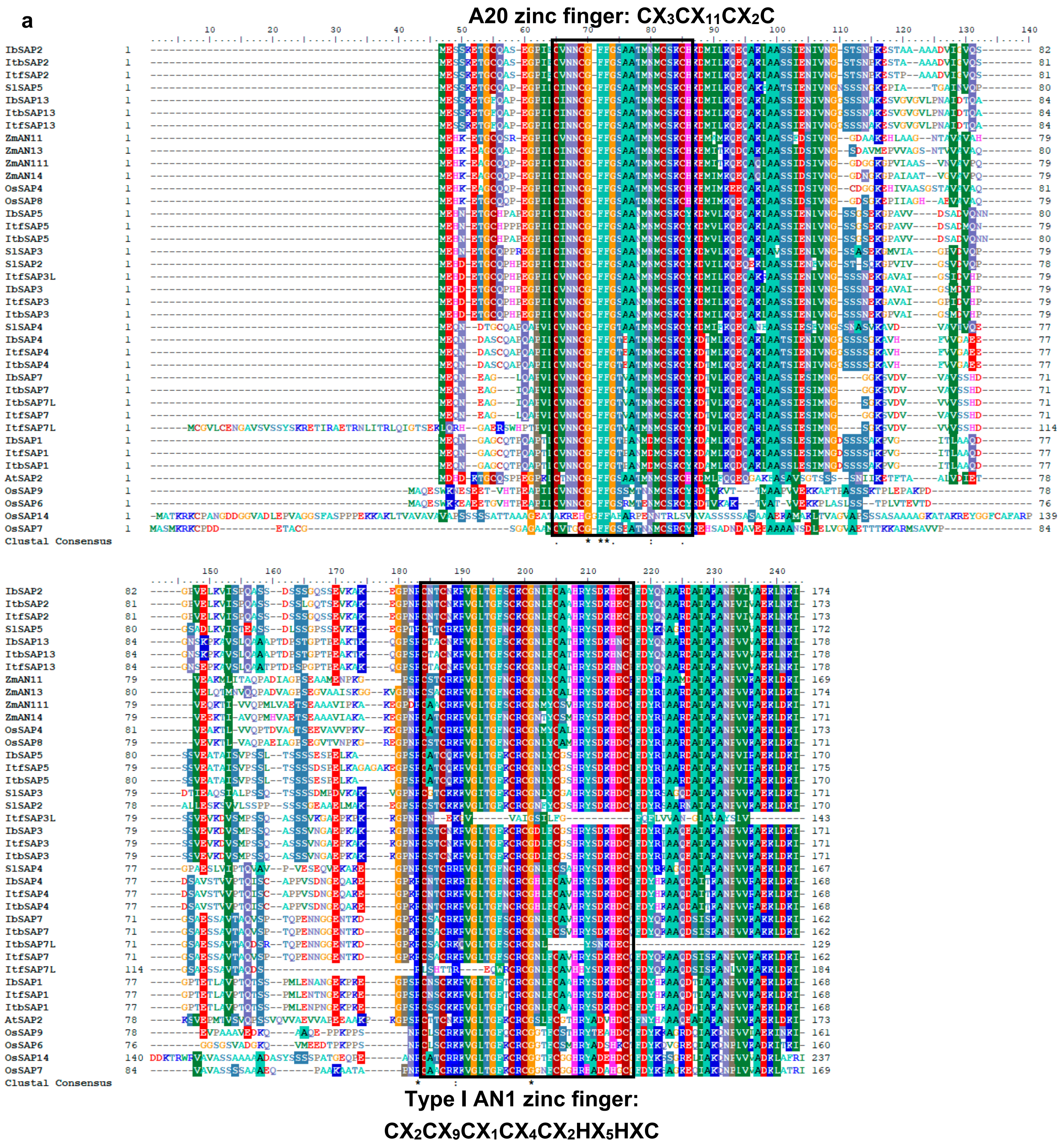

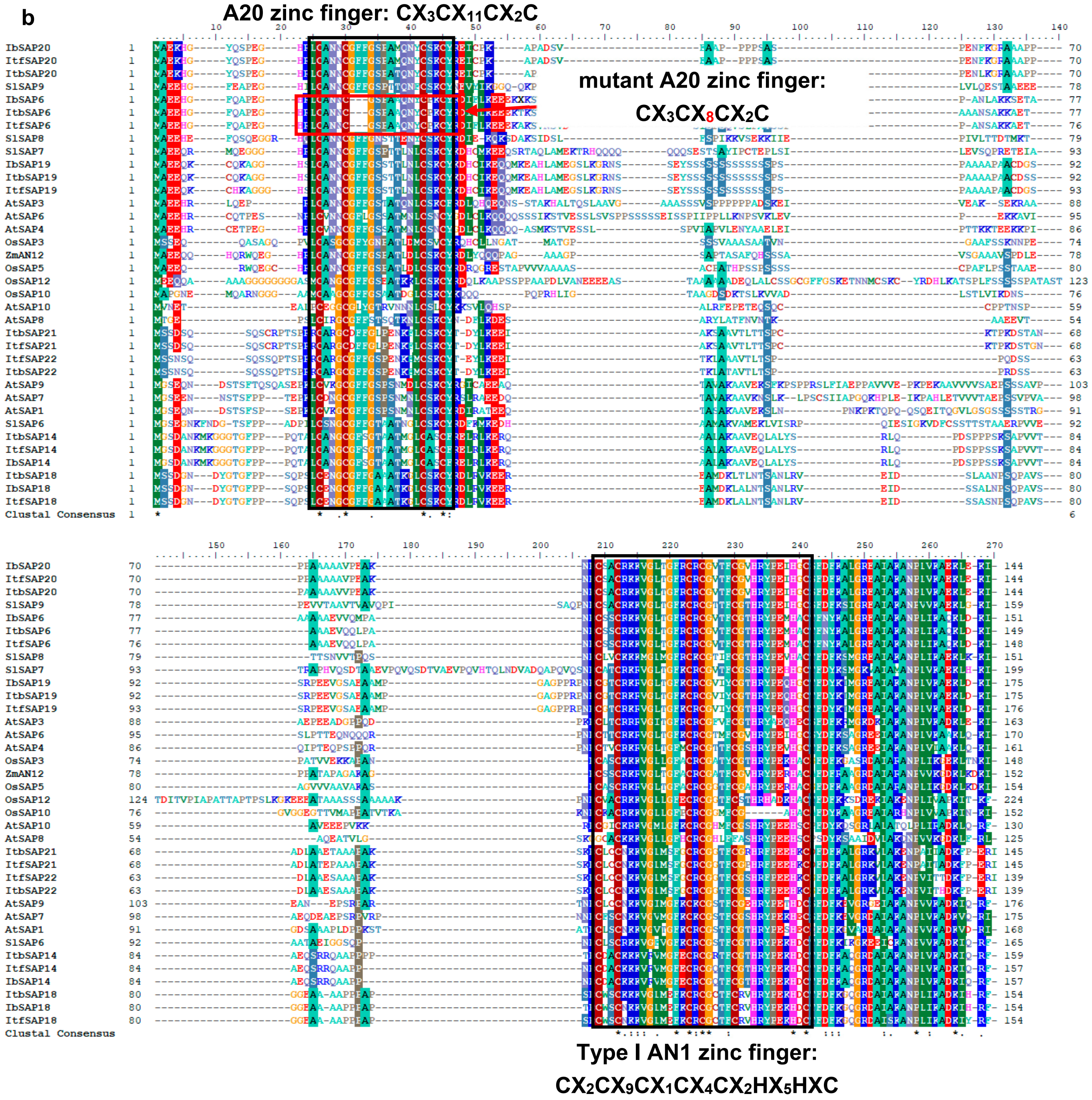
b
A20 zinc finger: CX3CX11CX2C
mutant A20 zinc finger: CX3CX8CX2C
Type I AN1 zinc finger: CX2CX9CX1CX4CX2HX5HXC
Clustal Consensus

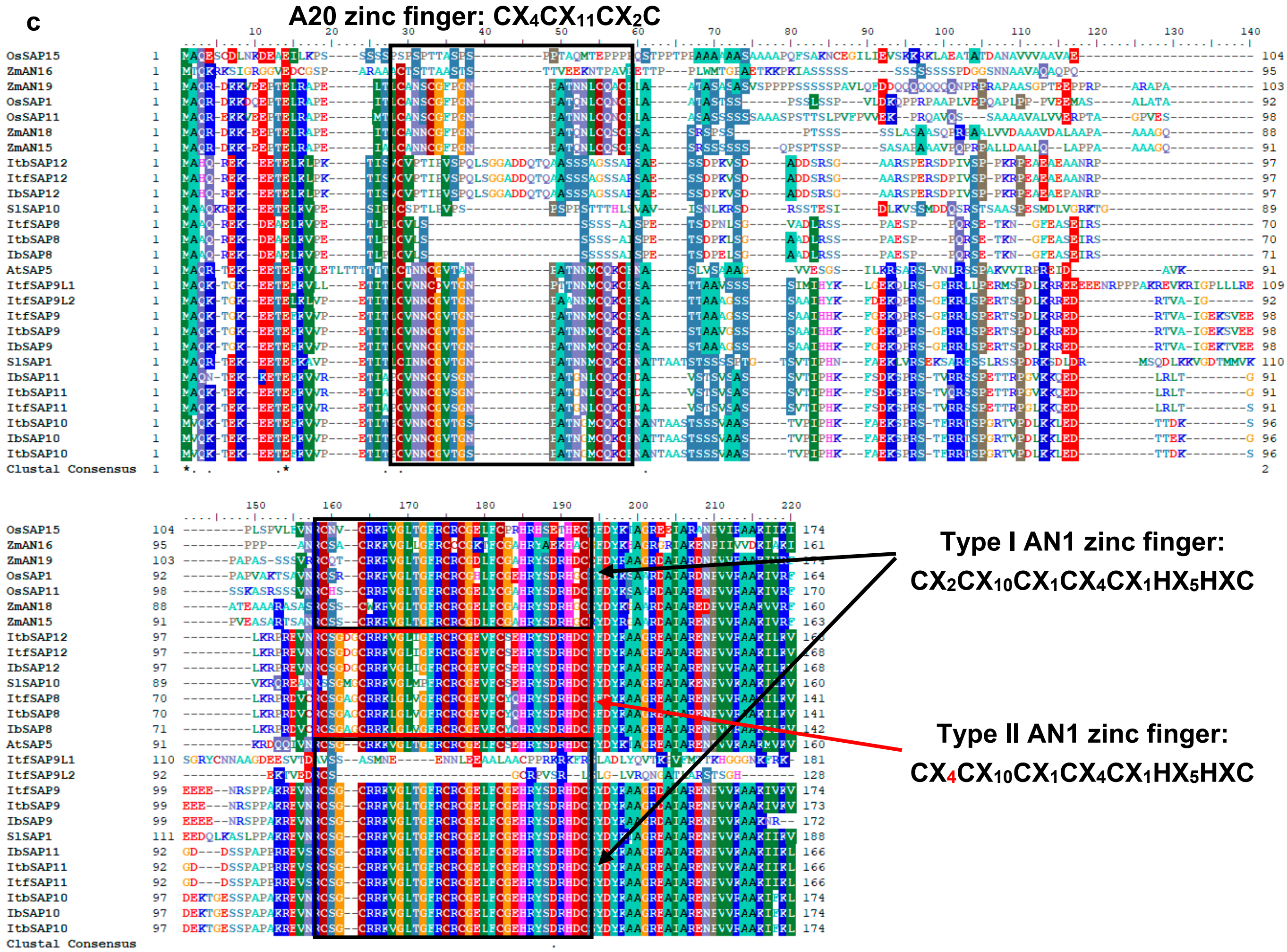
c
A20 zinc finger: CX4CX11CX2C
Type I AN1 zinc finger:
CX2CX10CX1CX4CX1HX5HXC
Type II AN1 zinc finger:
CX4CX10CX1CX4CX1HX5HXC

d

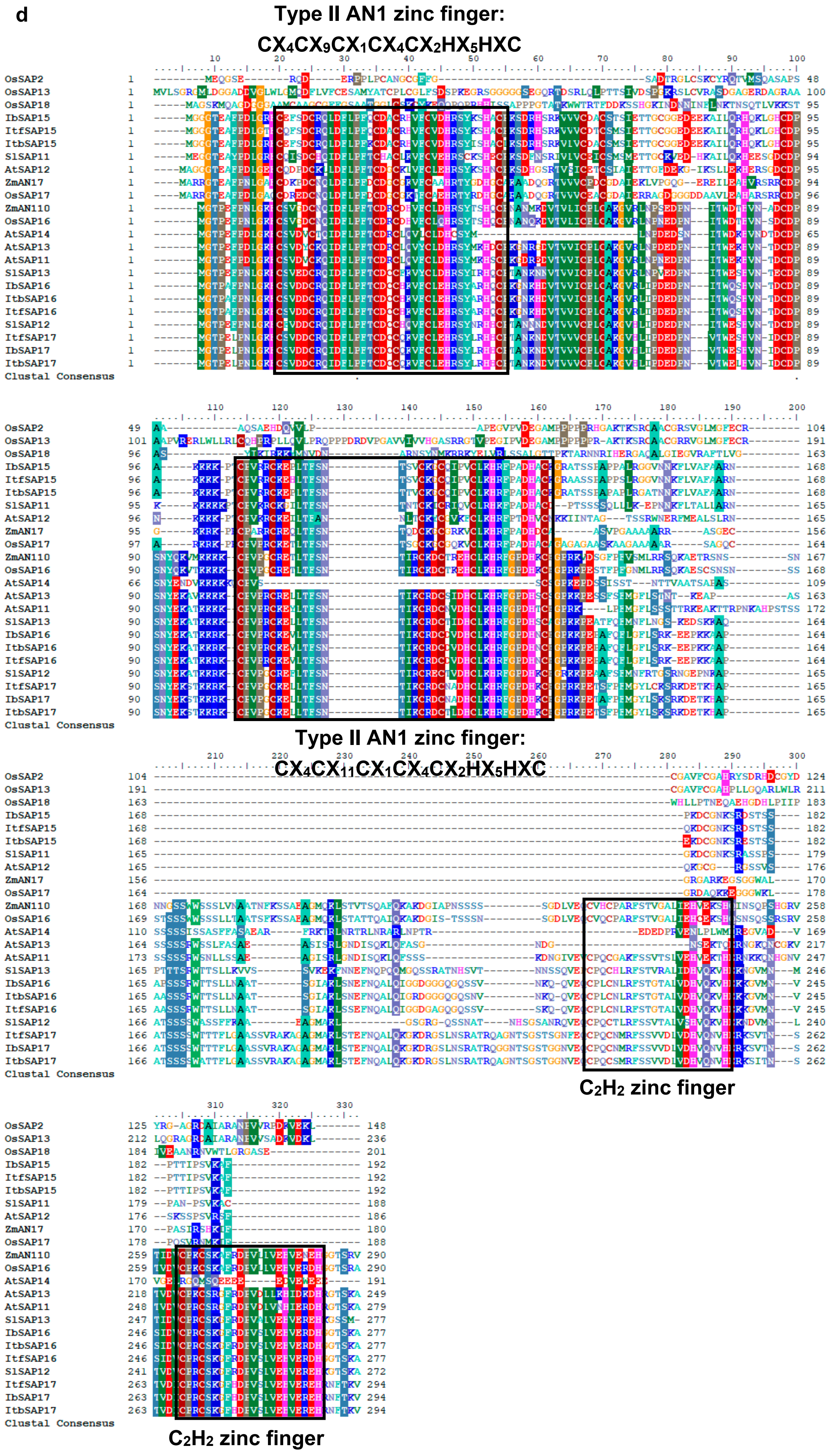
Type II AN1 zinc finger:
CX4CX9CX1CX4CX2HX5HXC
Type II AN1 zinc finger:
CX4CX11CX1CX4CX2HX5HXC
C2H2 zinc finger
C2H2 zinc finger
OsSAP2
OsSAP13
OsSAP18
IbSAP15
ItfSAP15
ItbSAP15
SlSAP11
AtSAP12
ZmAN17
OsSAP17
ZmAN110
OsSAP16
AtSAP14
AtSAP13
AtSAP11
SlSAP13
IbSAP16
ItbSAP16
ItfSAP16
SlSAP12
ItfSAP17
IbSAP17
ItbSAP17
Clustal Consensus

Figure S1. Alignment of amino acid sequences of clade I **(a)**, II **(b)**, III **(c)**, and IV **(d)** SAPs mentioned in this study. Amino acid sequences of SAPs were aligned and shown with ClustalW method in BioEdit program. A20 zinc fingers, type I AN1 zinc fingers, type II AN1 zinc fingers, and C_2H_2 zinc fingers were indicated with frames, respectively. "*" indicates positions which have a single, fully conserved residue. ":" indicates that one of the following 'strong' groups is fully conserved. "." indicates that one of the following 'weaker' groups is fully conserved.

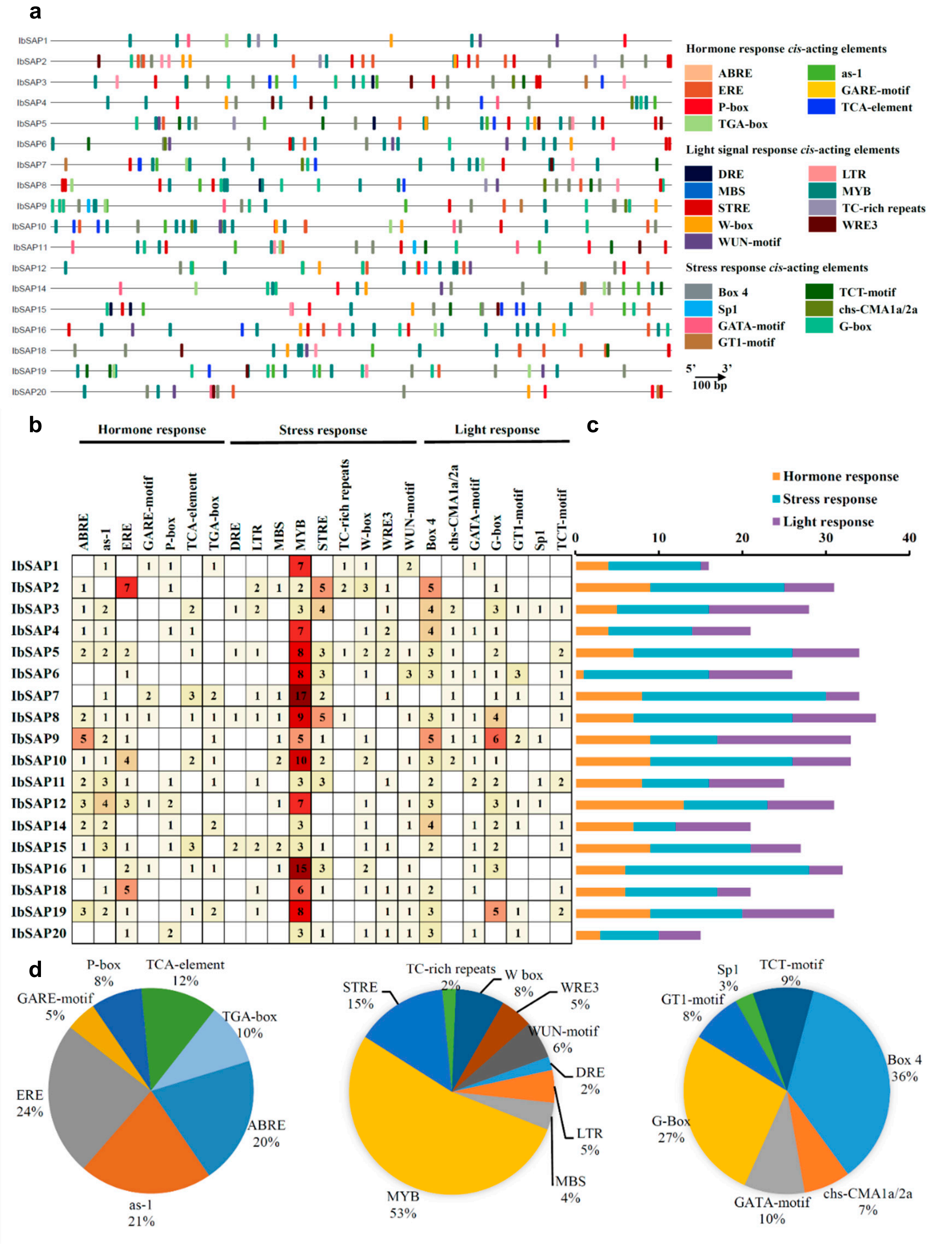

Figure S2. *Cis*-acting elements prediction in *IbSAP* promoters. 2,000 bp upstream sequences of *IbSAP*s were sent to PlantCARE for *cis*-acting element analysis. **a**, distribution of *cis*-element in *IbSAP* promoters. **b,** numbers indicate the number of each *cis*-acting element in the promoter of each *IbSAP* gene. **c**, numbers of hormone, light, and stress response *cis*-acting elements in the promoter of each *IbSAP* gene are shown. **d**, the percentage of different *cis*-acting elements in each element class.

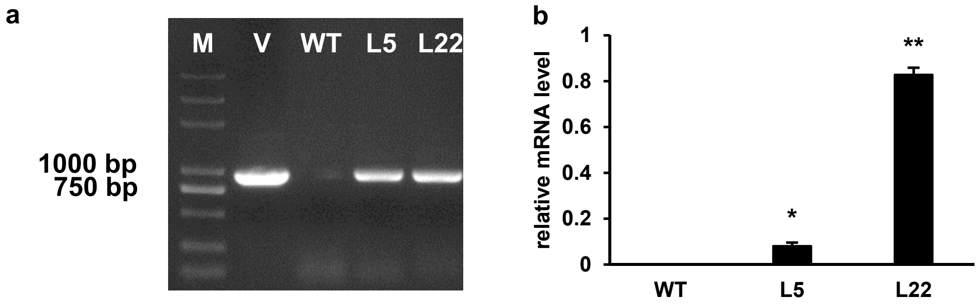

Figure S3. Identification of *IbSAP16*-transgenic *Arabidopsis*. **a**, genome PCR identification of *IbSAP16*-transgenic *Arabidopsis* lines. M, marker; V, pGWB12-*IbSAP16* vector control; WT, wild type *Arabidopsis*; L5 and L22, *IbSAP16*-transgenic *Arabidopsis* line 5 and 22. **b**, relative expression level of *IbSAP16* in WT and transgenic lines by RT-qPCR. The *Arabidopsis Actin2* gene was used as an internal reference. Data are presented as means ± standard error (n = 3). "*" or "**" indicate a significant difference from that of WT at $p < 0.05$ or 0.01, by Student's *t*-test.
